# Investigation of the awareness of and demand for hospice care and attitudes towards life-sustaining treatment at the end of life among community residents in Hangzhou

**DOI:** 10.1186/s12904-020-00628-8

**Published:** 2020-08-17

**Authors:** Yanhong Xie, Ying Xu, Shulan Yang, Jing Yan, Xiao Qing Jin, Caixia Liu

**Affiliations:** 1 Department of Geriatrics, Zhejiang Hospital, Hangzhou, 310013 Zhejiang Province China; 2 Department of Nursing, Zhejiang Hospital, Hangzhou, 310013 Zhejiang Province China; 3 Zhejiang Hospital, Hangzhou, 310013 Zhejiang Province China

**Keywords:** Hospice care, Cognition, End-of-life support therapy, Needs, Survey

## Abstract

**Background:**

To understand the status of residents’ awareness of and demand for hospice care services in Hangzhou and to provide a reference for promoting the formulation of hospice care-related policies in China.

**Methods:**

A small cross-sectional survey of 519 adults aged over 40 years old living in the rural-urban fringe and urban area of Xihu District, Hangzhou City, was conducted using convenience sampling and a self-designed questionnaire. The measures assessed awareness of hospice care (13-item scale), attitudes towards life support therapy (3-item scale), and demand for hospice care services (9-item scale).

**Results:**

The rate of awareness of hospice care among community residents was 50.30%. A total of 51.0% of residents wanted only comfortable life-sustaining treatment at the end of their lives. The acceptance of hospice care was positively correlated with the degree of understanding (x^2^ = 18.382, *P* = 0.001), and residents in the urban area were more likely to prefer hospice care than residents in the urban-rural fringe (x^2^ = 7.186, *P* = 0.028). Elderly residents showed a stronger tendency to prefer comfortable life support therapy (x^2^ = 12.988, *P* < 0.001). A total of 83.04% of the residents accepted the current necessity for hospice care to be provided in medical institutions. The preferred locations were professional hospice care institutions or general hospitals. A total of 93.64% of the residents agreed that the number of beds in hospice care wards should not exceed 2. In addition, the residents could afford part of the out-of-pocket expenses for hospice care services, with the ability to pay under 200 yuan per day, and the improvement of facilities was expected.

**Conclusions:**

To improve public awareness and acceptance of hospice care and promote healthy development in China, it is necessary to promote hospice care education for everyone.

## Background

Hospice care began in the 1960s. According to the WHO definition, hospice care focuses on improving the quality of life of patients and their families who face problems associated with life-threatening illness through the prevention and relief of suffering by means of the early identification and treatment of other problems, including physical, psychosocial and spiritual problems [1]. Although hospice care was introduced to mainland China as early as 1988, its acceptance in Chinese society has been relatively slow. An important question for patients and their families is whether to continue or forego treatment at the end of life. One study showed that people with and without advance directives (ADs) had different preferences concerning different treatments [2]. Another study [3] showed that the choice of life-sustaining therapy was related to the patient’s course and needs and that the decision to receive life-sustaining therapy differed according to differences in specific therapy measures.

With the aging of the population, the demand for care at the end of a disease is also increasing. According to an index system of the demand for hospice care (based on the burden caused by disease, dependency ratio of the elderly population, and speed of aging) [4], the objective demand in China is increasing.

At present, there are approximately 7.5 million deaths in China each year, and in approximately 80% of cases (6 million people), hospice care services are needed; however, the percentage of people receiving hospice care services in China lags significantly behind that in developed countries [5]. In October 2015, the Economist Intelligence Unit (EIU) released the Quality of Death Index for 2015; among 80 countries and regions in the world, Britain ranked first, and China ranked in the last 10 [6] This situation reflects the limited availability and low quality of palliative care in China. In recent years, with the promotion of the National Health Planning Commission, an increasing number of domestic scholars and policy makers have committed to “promoting the work of hospice care”. According to incomplete statistics, hospice services in China have achieved good social benefits. In 2018, a total of 283,000 end-of-life patients were served, and the coverage rate of hospice care services reached 4.7% [7]. The government’s policy support has just started, and residents’ awareness and needs are important factors affecting government decisions. Understanding public perceptions about hospice may also help inform public education efforts to improve awareness of hospice as an option at the end of life [8]. Previous studies showed that 86% of adults living in the contiguous US had heard about hospice care [8], 76.3% of respondents in Northern Ireland had heard the term palliative care [9], 93% of community-dwelling older adults were familiar with term “hospice” [10], and 60% of New York State residents associated hospice and palliative care with end-of-life care [11]. More than half (52.8%) of the participants of one study (Korean residents aged 60 and older in Tampa and Orlando) reported that they had heard about hospice, and 73.6% of the sample expressed a willingness to use hospice [12]. A total of 82% of community-dwelling older adults in America strongly agreed/agreed that “hospice can make people feel better” [10]. (62.9%) New York State residents were very likely to recommend this type of care to a loved one, while 224 (28%) were somewhat likely [11].

Before providing services in any area, it is necessary to estimate the needs of local residents [13]. Therefore, our study was based in Zhejiang Province. The investigation of community residents in the Hangzhou metropolitan area and an integrated urban-rural community increases our understanding of the status of the awareness of and demand for hospice care among residents of different occupations and educational levels and provides an empirical basis for the dissemination of information about hospice care, the popularization and development of hospice care services and the formulation of related policy.

## Methods

### Study design

This study used a cross-sectional design and a sample of 519 older adults, who completed a questionnaire via an in-person interview. Before the data collection, written informed consent in Chinese was obtained from the participants. Research protocols were approved by the Zhejiang Hospital Ethics Institutional Review Board (IRB).

### Sample, participants, setting, and recruitment

A convenience sampling method was used to select the participants. After IRB approval, participants were recruited from two sites, i.e., the urban-rural fringe and the metropolitan area of Hangzhou, from October 14 to November 8, 2018. The researchers defined pedestrians at the gate of the community as the survey objects. Pedestrians were invited to participate in the study via an announcement at the gate of the community and the distribution of flyers. After the announcement, adults who were interested in participating in the study were asked to provide their names and contact information by completing a sign-up sheet that would be passed around the dining hall or by contacting the researcher or research assistant to schedule an appointment. A total of 529 adults expressed an interest in participating in the study. Before scheduling an appointment for the interview, the participants were screened for eligibility for the study based on several criteria. One of the criteria concerned cognitive ability, which we assessed by using the Mini-Mental State Examination (MMSE) [14]. The MMSE covers the following seven aspects: time orientation, place orientation, immediate memory, attention and calculation, delayed memory, language, and visual space. For each of the 30 questions, 1 point is given for a correct answer, and 0 points are given for a wrong answer or unknown response; thus, the total score ranges from 0 to 30 points. The test scores are closely related to the level of education. The thresholds for normal cognition by education level have been defined as follows: illiterate, > 17 points; primary school, > 20 points; and junior high school and above, > 24 points. Other inclusion criteria were healthy adults from different families without cognitive impairment who had lived in Hangzhou for more than 2 years and were over 40 years old. The exclusion criteria were as follows: medical workers with hearing or cognitive impairment who refused to participate in the survey. All the respondents provided informed consent and volunteered to participate in the survey. Among the 530 persons who indicated an interest in participating in the study, 6 participants did not meet the eligibility criteria (4 due to cognitive ability and 2 due to hearing ability). Ultimately, 524 persons agreed to participate and signed the consent form.

Before the survey was conducted, the questionnaire content instructions were formulated; the investigators were trained uniformly; and the purpose, significance and points for attention of the survey were explained with uniform guidance. The questionnaire was completed anonymously by participants and then checked and collected by the investigators on the spot. The questionnaires were administered via face-to-face interviews at a community neighborhood committee office. Each interview lasted approximately 15 to 20 min, and respondents were offered a water bottle worth RMB 15–20 yuan as compensation. In this survey, 524 questionnaires were distributed. A total of 519 valid questionnaires were included in the analysis, while 5 questionnaires were considered invalid due to reasons such as the overselection of responses and missing responses; thus, the effective recovery rate was 99.05%.

### Questionnaire

A questionnaire on hospice care was designed by the project team based on the relevant literature [15, 16] and consultation with experts at home and abroad. The questionnaire consisted of four parts, including general information, hospice care cognition, acceptance of hospice care, and the demand for hospice care services. The general information questionnaire included 10 items covering information such as residence, gender, age, marital status, education level and monthly income. The second part assessed residents’ cognitive attitudes towards hospice care, including the level of knowledge of hospice care (5 items), understanding (1 item), perceived objective and purpose of hospice care (2 items), treatment meaning (1 item), attitudes toward death acceptance and illness notification (3 items), and the perceived reason for the lack of the sufficient popularity of hospice therapy (1 item). The level of knowledge of hospice care was evaluated with the question “Have you heard about hospice care?”, followed by 4 questions, each with one correct answer and several incorrect answers. If the respondent chose “have heard about it” and answered 3 or more questions correctly, he/she was considered to have a “better understanding”. If the respondent chose “have heard about it” and answered 1–2 questions correctly, he/she was considered to have a “basic understanding”. If the respondent chose “have never heard about it” or chose “have heard about it” and answered 0 questions correctly, he/she was considered to have “no understanding”. The third part investigated community residents’ acceptance of hospice care, including their willingness to sign a living will, their preference regarding medical decision-making, their willingness to accept hospice care services, etc. The fourth part assessed community residents’ demand for hospice care services, including their choice of the hospice service location, the facilities and staffing of service organization, the charging standards, and family members’ needs for physical and mental care during the patient’s terminal stage. After the questionnaire was developed, experts were consulted. The validity of the questionnaire was 0.92 according to the Delphi expert consultation method. Twenty-five residents were selected for to complete the questionnaire two times, with the retest conducted 2 weeks after the first test. The correlation coefficient of the two sets of results was 0.906 (*P* < 0.05), which indicated that the questionnaire had good test-retest reliability.

Life-sustaining treatment is defined as any medical treatment that is intended to help someone or something to stay alive; that is, it supports or extends life [17]. Active life-sustaining treatment refers to the administration of all measures to sustain life, such as cardiopulmonary resuscitation (CPR), mechanical or artificial ventilation, clinically assisted nutrition and hydration, circulatory support, blood purification, or antibiotics. Comfortable life-sustaining treatment refers to life-sustaining measures aimed at alleviating the suffering of patients and meeting their physiological and psychological needs.

### Statistical analysis

IBM SPSS vs. 25.0 software was used for statistical analysis. We calculated descriptive statistics for the entire sample. The chi-square test was used for categorical data analysis and group comparison. Continuous data were analyzed by the Kruskal-Wallis test. The association was considered statistically significant when *p*-values were less than 0.05.

## Results

### Characteristics of the participants

A total of 519 people responded, for a 99.05% response rate. As shown in Table 1, there were 269 residents in the rural-urban fringe zone, accounting for 51.83% of the sample, and the remaining 250 were urban residents, accounting for 48.17% of the sample. Among the respondents, 227 were males (43.74%), and 292 were females (56.26%). Regarding their ages, 82 respondents were aged 40–49, accounting for 15.80% of the sample; 108 were aged 50–59, accounting for 20.81%; 171 were aged 60–69, accounting for 32.95%; and 158 were aged ≥70, accounting for 30.44%. Regarding marital status, 65 (12.52%) people had no spouse, and 454 (87.48%) had spouses. In terms of education, 62 people had a primary school or below education level, accounting for 11.95% of the sample; 100 had a junior high school above education level, accounting for 19.27%; 149 had a senior high school or technical secondary school educational level, accounting for 28.71%; and 208 had higher education, accounting for 40.08%. Regarding income, 27 people had no monthly income, accounting for 5.20%; 99 people earned 1–3000 yuan, accounting for 19.08%; 212 people earned 3001–5000 yuan, accounting for 40.85%; 150 people earned 5001–10,000 yuan, accounting for 28.90%; and 31 earned more than 10,000 yuan, accounting for 5.97%. In regard to the medical system, 76 people had new rural cooperative medical insurance, accounting for 14.64%; 123 people had basic medical insurance for urban residents, accounting for 23.70%; 291 people had basic medical insurance for urban workers and residents, accounting for 56.07%; and 29 people had no medical insurance and paid out of pocket, accounting for 5.59%. In regard to religious beliefs, 365 people had no religious beliefs, accounting for 70.33%, and 154 people had religious beliefs, accounting for 29.67%. Moreover, 365 people had experience with death of immediate relatives, accounting for 70.33%. In addition, 253 people had experience caring for dying patients, accounting for 48.75%.

**Table 1:** Attitudes towards life-sustaining treatment at the end of life by participants’ demographic characteristics n (%)

Project	Category	Life-sustaining treatment attitudes at the end of life			Total	x _trend_^2^/x^2^	P‧
		Active treatment	Comfortable support treatment	No treatment needed	(*n* = 519)		
		(*n* = 181)	(*n* = 265)	(*n* = 73)			
Area of residence	Rural-urban fringe	108 (40.15)	124 (46.10)	37 (13.75)	269 (51.83)	7.186	0.028*
	Urban area	73 (29.20)	141 (56.40)	36 (14.40)	250 (48.17)		
Gender	Male	79 (34.80)	116 (51.10)	32 (14.10)	227 (43.74)	0.001	0.975
	Female	102 (34.93)	149 (51.03)	41 (14.04)	292 (56.26)		
Age	40 ~ 49	34 (41.46)	42 (51.22)	6 (7.32)	82 (15.80)	12.988	< 0.001c
	50 ~ 59	51 (47.22)	43 (39.81)	14 (12.96)	108 (20.81)		
	60 ~ 69	52 (30.41)	94 (54.97)	25 (14.62)	171 (32.95)		
	70 ~ 79	34 (30.09)	63 (55.75)	16 (14.16)	113 (21.77)		
	> 80	10 (22.22)	23 (51.11)	12 (26.67)	45 (8.67)		
Marital status	Single	22 (33.85)	34 (52.31)	9 (13.85)	65 (12.52)	0.048	0.976
	Married	159 (35.02)	231 (50.88)	64 (14.10)	454 (87.48)		
Education level	Primary school and below	28 (45.16)	25 (40.32)	9 (14.52)	62 (11.94)	2.677	0.102
	Junior	52 (34.90)	73 (48.99)	24 (16.11)	149 (28.71)		
	High School/Technical School	52 (35.37)	72 (48.98)	23 (15.65)	147 (28.32)		
	Junior college or above	60 (28.85)	123 (59.15)	25 (12.02)	208 (40.08)		
Monthly after-tax income (RMB)	No income	14 (51.85)	9 (33.33)	4 (14.81)	27 (5.20)	1.753	0.185
	1 ~ 3000	39 (39.39)	45 (45.45)	15 (15.15)	99 (19.08)		
	3001 ~ 5000	70 (33.02)	110 (51.89)	32 (15.09)	212 (40.85)		
	5001 ~ 10,000	51 (34.00)	83 (55.33)	16 (10.67)	150 (28.90)		
	> 10,000	7 (22.58)	18 (58.06)	6 (19.35)	31 (5.97)		
Medical insurance	Out of pocket	10 (34.48)	12 (41.38)	7 (24.14)	29 (5.59)	4.868	0.561
	New rural cooperative medical service	32 (42.11)	35 (46.05)	9 (11.84)	76 (14.64)		
	Basic medical insurance for urban residents	42 (34.15)	64 (52.03)	17 (13.82)	123 (23.70)		
	Basic medical insurance for urban workers	97 (33.33)	154 (52.92)	40 (13.75)	291 (56.07)		
Religious beliefs	No	27 (7.40)	148 (40.55)	190 (52.05)	365 (70.33)	1.759^a^	0.079
	Yes	16 (10.40)	70 (45.50)	68 (44.20)	154 (29.67)		
Experience of the death of an immediately family member	Yes	127 (34.79)	190 (52.05)	48 (13.15)	365 (70.33)	0.973	0.615
	No	54 (35.06)	75 (48.70)	25 (16.23)	154 (29.67)		
Experience of caring for dying patients	Yes	95 (37.55)	127 (50.20)	31 (12.25)	253 (48.75)	2.237	0.327
	No	86 (32.33)	138 (51.88)	42 (15.79)	266 (51.25)		

### Current situation of community residents’ awareness of hospice care

The survey found that 8.29% of the residents reported better knowledge of hospice care and that 42.00% reported a basic knowledge; that is, 50.30% knew about hospice care. A total of 46.82% did not know about hospice care, and 2.89% did not want to know about hospice care and believed there was a taboo surrounding it. As we can see in Table 2.

**Table 2:** The relationship between community residents’ awareness of hospice care and their attitudes towards life-sustaining treatment

Project	Category	Life-sustaining treatment attitudes at the end of life			Total (*n* = 519)	x^2^	P‧
		Active treatment (*n* = 181)	Comfortable support treatment (*n* = 265)	No treatment needed (*n* = 73)			
Awareness of hospice care	Better understanding	11 (25.58)	29 (67.44)	3 (6.98)	43 (8.29)	18.382	0.001**
	Basic understanding	61 (27.98)	127 (58.26)	30 (13.76)	218 (42.00)		
	No understanding	109 (42.24)	109 (42.25)	40 (15.50)	258 (49.71)		
Total		181 (34.88)	265 (51.05)	73 (14.06)	519 (100.00)		

### Community residents’ attitudes towards life-sustaining treatment at the end of life

In addition, 34.9% of the residents preferred active life-sustaining treatment, 51.0% of the residents wanted only noninvasive, comfortable life-sustaining treatment, and 14.1% of the residents did not think they needed any life-sustaining treatment. As we can see Table 2.

### The relationship between community residents’ awareness of hospice care and their attitudes towards life support therapy

The chi-square results showed that the awareness of hospice therapy and nursing care was related to the choice of life support therapy. The greater the patient’s understanding of hospice therapy and nursing care, the more inclined the patient was to choose comfortable life support therapy (x^2^ = 18.382, *P* = 0.001). The results are shown in Table 2.

### Attitudes towards life-sustaining treatment at the end of life by participants’ demographic characteristics

According to Table 1, there were significant differences in the demand for life support therapy among residents from different areas of residence and of different ages (*P* < 0.05), while there were no differences based on other categories such as gender, marital status, education, monthly after-tax income, medical insurance type, religious beliefs, experience of the death of immediate relatives and experience of the care of dying patients (*P* > 0.05). Furthermore, based on a comparison of the percentage differences, residents of the metropolitan area were more likely to prefer noninvasive and comfortable life-sustaining treatment (x^2^ = 7.186, *P* < 0.05) residents of the urban-rural fringe. In addition, the chi-square for trend showed that age was related to the choice of life support therapy. The older the patient’s age, the more choice likely he or she was to indicate a preference for comfortable life support therapy or no need for life support therapy (x^2^_trend_ = 12.988, *P* < 0.01).

### Community demand for hospice care services

According to Tables 3 and 4, community residents’ main expectations of hospice care were reduced discomfort symptoms (such as pain and nausea) and side effects of drugs (75.53%), medical help (60.31%) and companionship (50.1%). A total of 83.04% of the community residents believed that it was necessary for medical institutions to provide hospice care. A total of 49.71 and 44.32% of the residents wanted to receive hospice care services in professional hospice care institutions or general hospitals, respectively, and 39.69% of the residents wanted to receive hospice care services at home. The proportions of residents who were willing to accept hospice care in nursing institutions or community health centers were 39.50 and 21.97%, respectively. A total of 62.43% of the residents desired the average daily out-of-pocket costs of hospice care service to be less than 200 yuan, and more than 80% of the residents (87.48%) believed that hospice care drugs and services should be included in medical insurance reimbursement. A total of 85.55% of the residents thought that the hospital should establish a separate hospice ward, 88.82% of the residents agreed that the hospice ward should have a family waiting room, 82.27% of the residents thought that the hospice ward should have special equipment (automatic bathing machine, automatic turning over bed, etc.), and 83.04% of the residents thought that the hospice ward should have a farewell room/caring room.

**Table 3:** Community demand for hospice care services, n (%)

	Service expectations (multiple choice)			Preferred location (multiple choice)			Expectations about number of beds per room			Out-of-pocket costs (RMB/month)		
	Expectation	Frequency	Percentage	Location	Frequency	Percentage	Number of beds	Frequency	Percentage	Cost	Frequency	Percentage
1	Reduced discomfort	392	75.53	Professional hospice care institutions	258	49.71	One	234	45.09	< 200	324	62.43
2	Medical help	313	60.31	General hospital	230	44.32	Two	252	48.55	200 ~ 400	138	26.59
3	Company	260	50.10	Home	206	39.69	Three	27	5.2	400 ~ 600	32	6.17
4	Assistance in completing a will	214	41.23	Pension agency	205	39.5	Other	6	1.16	600 ~ 800	12	2.31
5	Funeral preparations	177	34.1	Community health center	114	21.97				> 800	13	2.50

**Table 4:** Other resident needs for hospice care services, n (%)

Need (multiple choice)	Frequency‧	Percentage‧
Hospice care is now necessary in medical institutions	431	83.04
Hospice care drugs and services should be covered by medical insurance reimbursement	454	87.48
Hospice patients should be accompanied by family members	461	88.82
Special equipment is needed (automatic bathing machine, automatic turning bed, etc.)	427	82.27
A farewell room/care room should be provided	431	83.04

## Discussion

This study aimed to understand the status of residents’ awareness of and demand for hospice care services in Hangzhou and to provide a reference for promoting the formulation of hospice care-related policies in China. The results showed that the rate of awareness of hospice care among community residents was 50.30%. A total of 51.1% of residents desired comfortable life-sustaining treatment at the end of their lives. The acceptance of hospice care was positively correlated with degree of understanding. Residents of metropolitan areas were more likely to prefer hospice care than residents of urban-rural areas, and elderly residents showed a stronger preference for comfortable life support therapy. A total of 83.04% of the residents accepted the current necessity for hospice care to be provided in medical institutions, and the preferred locations for hospice care were professional hospice care institutions or general hospitals. A total of 93.64% of the residents agreed that the number of beds in hospice care wards should not exceed 2. In addition, the residents could afford to pay part, i.e., below 200 yuan per day, of the out-of-pocket expenses for hospice care services, and the improvement of facilities was expected.

### Community residents have low awareness of hospice care

The survey showed that the rate of awareness of hospice care among community residents was 50.30%, which indicated that residents had a certain degree of understanding of hospice care. This result was similar to that of Tang Zheng yan’s survey of community residents in Xi’an (51.7%) and slightly higher than that reported in Sweden (43%) [18]. However, there is a large gap between the observed public awareness rate and those of advanced developed countries and regions in the world, such as Northern Ireland (the UK), at 81.0% [9] and the contiguous United States among the general population (86%) [8] and Hispanics (56%) [19] this gap is mainly related to the cultural background of our country. Influenced by our traditional culture, most people find talking about death to be taboo and generally still do not accept the concept of a “good death”. Therefore, the acceptance of hospice care services in Chinese society is relatively low. Until now, most medical resources in the general environment have been accepted slowly. These resources all focus on curative treatment. Due to the refusal to discuss topics such as death, China has been slow to embrace the concept of palliative care, in large part due to the lack of life education. In addition, the lack of support at the national level for law and policy on hospice care; the lack of positive propaganda and guidance from public opinion and the media on hospice care concepts, even in medical colleges and universities; and the lack of propaganda and education on death, hospice care and hospice care have led to a lack of understanding of hospice care among Chinese people. In addition, because China has adhered to the tradition of filial piety since ancient times and because of the tenets of filial piety, people think it is in violation of this principle to send elderly people to hospice care institutions, which invisibly hinders acceptance of the concept of hospice care.

### Community residents have demand for hospice care

Palliative and hospice care have been associated with improved patient symptom control and quality of life [20] as well as increased satisfaction with care. According to the present survey, 34.9% of the residents needed active life-sustaining treatment at the end of their lives. This may be related to the importance of the culture of filial piety in China. According to the available literature, a combination of race, individual characteristics, attitudes, beliefs, and behaviors contribute to a person’s preference for hospice [21]. When patients lose their decision-making ability, many family members fail to consider the advantages and disadvantages of life-sustaining treatment at the end of patients’ lives and instead hold onto the idea of saving the patient’s life, making rescue efforts ineffective and leading to a refusal to stop life-sustaining until the patient is exhausted. The proportion of residents willing to accept noninvasive and comfortable life-sustaining treatment was 51.1%, which was similar to the residents’ rate of awareness of hospice care (50.30%). Residents have demands or potential demands for hospice care services, and residents hope that hospice care services will mainly reduce their discomfort symptoms (75.53%) and provide medical help (60.31%) and companionship (50.10%).

### Improving the understanding of hospice is conducive to the development of hospice care

Despite the documented benefits of palliative and hospice care in improving patients’ quality of life, these services remain underutilized. Multiple factors limit the utilization of these services, including patients’ and caregivers’ lack of knowledge and misperceptions [11]. The evidence shows that public awareness campaigns can improve the awareness of palliative care and probably improve the quality of care [22]. The statistical analysis showed that the level of understanding of hospice care affected the acceptance of hospice care. The more people knew about hospice care, the more likely they were to accept hospice care, which was consistent with the results of Cagle et al. [8]. Recent research [23] has observed that the public has poor knowledge and awareness about hospice care, and several misperceptions exist; these findings have remained constant over time despite growth in the field of hospice care, which highlights the strong need for focused educational interventions. Therefore, in the future, we should pay attention to the dissemination of hospice care knowledge and propaganda to allow more residents to learn about hospice care, which would be conducive to the development of hospice care.

### Different groups have different demands for hospice care

The study found that residents of metropolitan areas were more likely than those in the rural-urban fringe to prefer noninvasive and comfortable life support therapy. Respondents who were more educated were also more likely to be more knowledgeable about hospice [8]. This finding may be due to the relatively limited exposure to information about hospice among residents among residents of the rural-urban fringe, who hold relatively conservative views. Urban residents could more easily access information through newspapers, social networks, television and other channels and thus more readily accepted new ideas. They payed attention to eugenics but also had begun to pay attention to a ‘good death’. In addition, older people tended to choose comfortable life support therapy, which was consistent with the findings of Zhang et al. [24] and Cagle et al. [8]. Similarly, a previous research [25] result showed that older adults with higher education levels have greater odds of agreeing that advance directives are necessary. This may be because elderly people have experienced more deaths and have greater awareness that ineffective medical treatment not only increases the financial burden of their children but also may not improve their quality of life. Therefore, elderly people had a higher acceptance rate of hospice care.

### Hospice care in professional hospice care institutions and general hospitals is more acceptable than that offered in other locations

Dying in a preferred place is important for achieving a good death for both the patient and his/her family. Nearly half of the respondents hoped to receive hospice care in professional hospice institutions or general hospitals, while home (39.69%) was the third choice. This was inconsistent with the finding on the preferred place for hospice care in Northern Ireland (UK), which was family residence (61%) [9], and in Japan, where residents generally preferred hospice care at home (64%) [26]. The survey showed that approximately 70% of respondents chose home as the preferred place for EOL care and/or death [18]. Home was the most preferred place of death (56%), and the hospital was least preferred (25%) [27]. The reasons may be related to the lack of knowledge and awareness of hospice care among Chinese residents, the lack of a hospice care system and the singularity of hospice care services in China. In addition, Chinese people have more taboos about dying at home. In China, residents can obtain effective pain relief measures in hospitals, whereas home care is very limited.

A total of 83.04% of the residents believed that it was necessary for medical institutions to provide hospice care, which indicated that residents still had relative trust in hospitals. The reasons were as follows. (1) Residents believed that professional hospice and general hospitals had the necessary basic conditions and relatively mature care technology to provide hospice care, in contrast to community hospitals and pension institutions, and that the caregivers were more professional. The patients also felt more comfortable staying in hospitals. With families becoming increasingly younger and the increasing pressure of social competition, many families feel powerless or confused when their elderly relatives are dying. Therefore, it is necessary for professional hospice care institutions or general hospitals to provide hospice care to relieve the suffering of patients and reduce the burden of family members. It can be seen that in China, general hospitals will assume the main responsibility of hospice care for dying patients, and the promotion of hospice care needs to start from general hospitals.

### Other demands of residents for hospice care

According to the survey, the majority of residents (93.64%) agreed that the number of beds in the hospice ward should not exceed 2. With the development of China’s economy, residents’ expectations of quality of life are increasing. The past large-scale ward model of 4–6 people is not suitable given the current development of residents’ demands for hospice care. The affordability of hospice care cannot be separated from the coverage of universal health insurance. Countries with high death quality, such as Britain, Singapore and Japan, pay for hospice care services through universal health insurance. The results of this survey showed that 62.43% of the residents in the studied province wanted to take care of their own expenses for hospice care services, and more than 80% of them preferred to pay less than 200 yuan/day. Medicines and services should be included in medical insurance reimbursement, which suggests that hospice care should be gradually included in the scope of basic medical security in China. In addition, more than 80% of the residents hoped that the hospice and nursing institutions would have single rooms that allowed family to accompany the patient and farewell rooms/caring rooms. They also hoped that hospice and nursing institutions would be equipped with special equipment (such as automatic bathing machines and automatic patient-turning beds). This suggests that our country still needs to make efforts to improve the facilities of hospice and nursing service institutions.

## Conclusions

This study also has several potential limitations. This study used a convenience sampling method with a small sample size, which limits the generalizability of the results. People who did not volunteer to participate in the study might have had different characteristics, such as poor health and mental health status, than the included participants and thus might have had different perspectives on awareness and demand for hospice care. Future studies should employ a probability sampling method with a larger sample size to enhance generalizability. Second, the study population was over 40 years old, which potentially limits the generalizability of our findings to all adults. Finally, this study had a relatively small sample, therefore may limit broader inferences from the results. In the future, the geographical scope of the area from which respondents are sampled needs to be further expanded.

This study investigated the current situation of residents’ awareness of and demand for hospice care in Hangzhou. More than 50% of the residents knew about hospice care, and more than 50% of the residents were willing to receive hospice care at the end of their lives. Moreover, the greater residents’ understanding of hospice care was, the greater their acceptance; in addition, residents of the metropolitan area and elderly residents were more likely to choose comfortable life-sustaining treatment. Residents preferred hospice care to be provided in professional hospice care institutions and general hospitals. Most residents agreed that the number of beds in hospice care wards should not exceed 2. In addition, residents hoped that the out-of-pocket costs of hospice care services would be less than 200 yuan/day, and they expected the facilities of hospice and nursing service institutions to be improved. Therefore, it is necessary to provide hospice care education for everyone to improve public awareness and acceptance of hospice care in China. The results of the survey provide a reference for promoting the formulation of relevant policies on hospice care in China.

## Data Availability

The datasets used and/or analyzed during the current study are available from the corresponding author from bona fide researchers with appropriate ethical clearance.

## Abbreviations

EIU: Economist Intelligence Unit
